# Mechanisms of Axon Elongation Following CNS Injury: What Is Happening at the Axon Tip?

**DOI:** 10.3389/fncel.2020.00177

**Published:** 2020-07-03

**Authors:** William Rodemer, Gianluca Gallo, Michael E. Selzer

**Affiliations:** ^1^Shriners Hospitals Pediatric Research Center, Lewis Katz School of Medicine, Temple University, Philadelphia, PA, United States; ^2^Department of Anatomy and Cell Biology, Lewis Katz School of Medicine, Temple University, Philadelphia, PA, United States; ^3^Department of Neurology, Lewis Katz School of Medicine, Temple University, Philadelphia, PA, United States

**Keywords:** spinal cord injury, axon regeneration, cytoskeletal dynamics, neurofilaments, microtubules, actin, growth cone

## Abstract

After an injury to the central nervous system (CNS), functional recovery is limited by the inability of severed axons to regenerate and form functional connections with appropriate target neurons beyond the injury. Despite tremendous advances in our understanding of the mechanisms of axon growth, and of the inhibitory factors in the injured CNS that prevent it, disappointingly little progress has been made in restoring function to human patients with CNS injuries, such as spinal cord injury (SCI), through regenerative therapies. Clearly, the large number of overlapping neuron-intrinsic and -extrinsic growth-inhibitory factors attenuates the benefit of neutralizing any one target. More daunting is the distances human axons would have to regenerate to reach some threshold number of target neurons, e.g., those that occupy one complete spinal segment, compared to the distances required in most experimental models, such as mice and rats. However, the difficulties inherent in studying mechanisms of axon regeneration in the mature CNS *in vivo* have caused researchers to rely heavily on extrapolation from studies of axon regeneration in peripheral nerve, or of growth cone-mediated axon development *in vitro* and *in vivo*. Unfortunately, evidence from several animal models, including the transected lamprey spinal cord, has suggested important differences between regeneration of mature CNS axons and growth of axons in peripheral nerve, or during embryonic development. Specifically, long-distance regeneration of severed axons may not involve the actin-myosin molecular motors that guide embryonic growth cones in developing axons. Rather, non-growth cone-mediated axon elongation may be required to propel injured axons in the mature CNS. If so, it may be necessary to use other experimental models to promote regeneration that is sufficient to contact a critical number of target neurons distal to a CNS lesion. This review examines the cytoskeletal underpinnings of axon growth, focusing on the elongating axon tip, to gain insights into how CNS axons respond to injury, and how this might affect the development of regenerative therapies for SCI and other CNS injuries.

## Introduction

**Figure 1:**
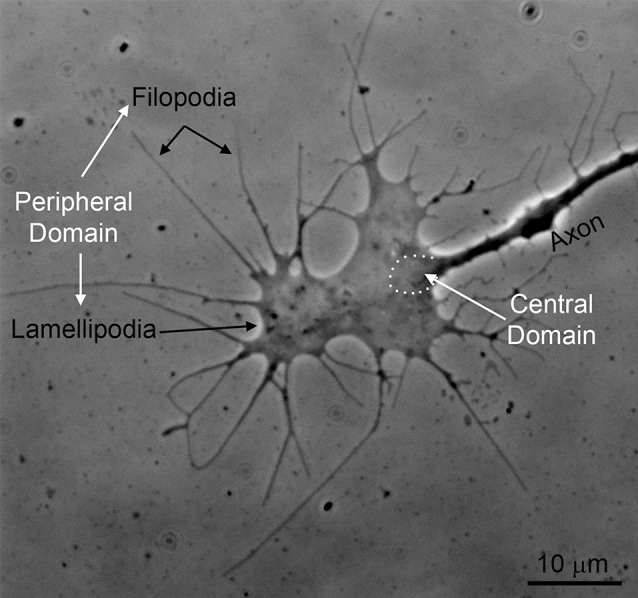
An example of the growth cone of a chicken embryonic sensory axon *in vitro* (phase-contrast imaging). The peripheral domain of growth cones consists of filopodia and flat lamellipodia. The central domain of growth cones is the region where the axon shaft dilates giving rise to the main body of the growth cone (approximated by the white dots). The central domain contains most of the organelles found in growth cones and the plus tips of axonal microtubules. The peripheral domain is supported by an underlying actin filament cytoskeleton.

Traumatic spinal cord injury (SCI) leads to devastating and persistent functional loss because damaged mammalian central nervous system (CNS) axons typically fail to regenerate. To restore lost function, injured axons must extend processes across various distances to reconnect with distal targets or form synaptic relays with interneuron populations. The growth cone, a specialized sensory-motility structure characterized by its distinctive distribution of actin, microtubule, and neurofilament cytoskeletal proteins, is the site of tip-mediated axon extension during development (Dent and Gertler, 2003; Figure 1). While the role of the growth cone in developmental axon extension has been studied extensively, its role in axon growth in response to CNS injury remains an active area of investigation. This review will focus on the cytoskeletal dynamics at the axon tip underlying regenerative axon extension.

Immediately following traumatic injury, the first task of the severed axon is to repair the axolemmal membrane to restore homeostasis and limit the influx of toxic factors from the extracellular environment. Membrane repair is an active, calcium-driven, proteolytic process that exploits the machinery of synaptic fusion to form a vesicle plug (Strautman et al., 1990; Spira et al., 1993; Steinhardt et al., 1994; Ziv and Spira, 1995; Howard et al., 1999; Spaeth et al., 2012; Zuzek et al., 2013). Importantly, axon resealing is not an all or nothing process but proceeds progressively as the vesicle plug stabilizes, and increasingly smaller molecules are excluded from the injured tip (Eddleman et al., 2000; Lichstein et al., 2000). Evidence from *in vitro* studies suggests the initial plug typically forms within 10–30 min after injury (Shi et al., 2000; McGill et al., 2016). However, the specific kinetics of resealing ultimately depends on multiple factors including species, neuron-type, axon caliber, and distance to the axon injury from the soma (McGill et al., 2016; Zhang et al., 2018). The calcium-dependent proteolytic environment that drives membrane resealing, in turn, is responsible for facilitating the dramatic cytoskeletal depolymerization and subsequent repolymerization needed to form a growth cone (Ziv and Spira, 1998; Bradke et al., 2012).

## Actin

**Figure 2:**
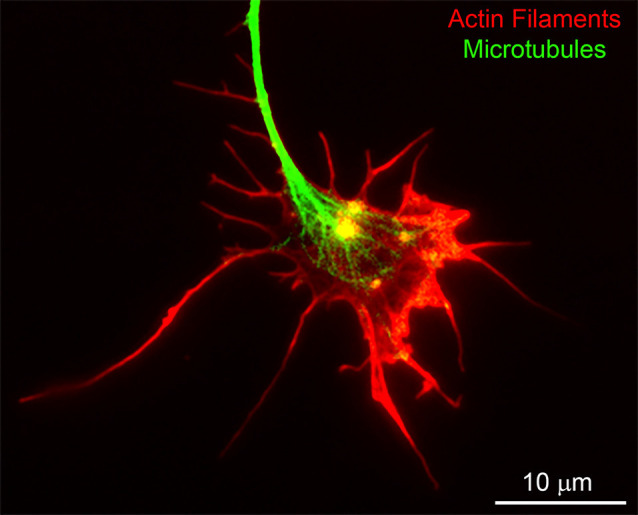
An example of the cytoskeleton of the chicken embryonic sensory axon growth cone. Actin filaments and microtubules were labeled using rhodamine-conjugated phalloidin and fluorescein-conjugated anti-alpha tubulin antibodies, respectively. Bundles of aligned actin filaments form the core of filopodia and meshworks of filaments support lamellipodia. The plus tips of axonal microtubules splay apart as they enter the central domain of the growth cone.

Growth cones are characterized by the elaboration of filopodia and lamellipodia; protrusive structures strictly dependent on actin filament nucleation, polymerization, and turnover (Figure 2). Although growth cones are required for axon guidance, they are not necessarily required for axon extension (Letourneau et al., 1987; Dent and Gertler, 2003). The inhibition of the extension of axons from cultured cerebellar neurons in response to actin filament depolymerizing drugs that collapse growth cones is dependent on the culturing substratum (Abosch and Lagenaur, 1993). Actin filament depolymerization does not impact axon extension on substrata coated with cell adhesion molecules such as L1 or P84, but strongly decreases extension on laminin and N-CAM. Embryonic sensory axons *in vitro* exhibit a developmental stage dependence for actin filaments, and thus growth cones, in maintaining some level of axon extension (Jones et al., 2006). Depolymerization of actin filaments in cultured hippocampal neurons does not impair the formation of minor processes, and axons are longer and extend at elevated rates in the presence of actin filament inhibitors (Ruthel and Hollenbeck, 2000). However, these findings have been challenged by reports suggesting that those inhibitors, namely cytochalasin E, reduced but did not completely abolish F-actin assembly (Chia et al., 2016). Ultimately, these studies indicate that the requirement for growth cones and the actin filament cytoskeleton in regulating the rate of axon extension is a complex issue and dependent on both neuron-intrinsic and extrinsic factors. Whether the growing tip of an axon should be called a “growth cone” despite the absence of filopodia, lamellipodia or an actin filament cytoskeleton is a semantic point, but because axon growth under different conditions may employ different mechanisms, we refer to simple-looking ends of axons that are growing without a prominent actin filament cytoskeleton as “growing axon tips.”

The vertebrate central nervous system undergoes a developmental transition from being able to regenerate axons to failing to regenerate. Extrinsic and intrinsic factors are thought to contribute to this transition (Tedeschi and Bradke, 2017). Herein we focus on neuron-intrinsic factors related to the cytoskeleton. The shapes of the growth cones of a variety of neurons undergo developmental simplification *in vivo* (Mason, 1985; Nordlander, 1987; Gorgels, 1991). This phenomenon also has been established *in vitro* by comparing the growth cones of the same population of neurons cultured from different developmental ages (Argiro et al., 1984; Kleitman and Johnson, 1989; Jones et al., 2006). The rates of filopodia elongation at growth cones, which are considered to reflect net polymerization of actin filaments at the tips of filopodia, also decrease with the age of neurons (Argiro et al., 1985). Dorsal root ganglion sensory neurons can be cultured from any age animal. *in vitro* studies have determined that as the sensory neuron ages, it undergoes a transition from forming one or two axons, which then grow rapidly, to generating multiple shorter axons, further emphasizing the intrinsic changes in axons and growth cones as neurons follow a developmental program (Smith and Skene, 1997). This program can be reversed by subjecting the axons of the sensory neurons to a “conditioning lesion” before culturing, indicating that the sensory neuron can revert to an earlier developmental stage of axon growth (Neumann and Woolf, 1999).

Growth Associated Protein 43 (GAP-43) is an important regulator of growth cone elaboration, acting through the regulation of the actin filament cytoskeleton (Denny, 2006). The levels of neuronal GAP-43 decline with developmental age (Jacobson et al., 1986). A conditioning injury to a peripheral nerve before a subsequent injury results in increased expression of GAP-43 and promotes sensory axon regeneration (Van der Zee et al., 1989; Cafferty et al., 2004). Overexpression of GAP-43 and the related CAP-23 in adult sensory neurons promotes sensory axon regeneration in the spinal cord (Bomze et al., 2001). GAP-43 thus provides an example of how a regulator of the actin filament cytoskeleton of growth cones undergoes developmental downregulation that correlates with decreased regenerative potential.

Cofilin and the related Actin Depolymerizing Factor (ADF) regulate actin filament turnover by accelerating the rate of filament severing, thus promoting the depolymerization of actin monomers from the pointed ends of filaments (Fass et al., 2004; Tanaka et al., 2018). A conditioning lesion increases the activation of cofilin and cofilin is required for the promotion of axon regeneration by the conditioning lesion (Tedeschi et al., 2019). Overexpression of cofilin in non-injury-conditioned neurons also promotes dorsal column axon regeneration after an SCI (Tedeschi et al., 2019). As determined by point mutants of cofilin, the actin severing activity of cofilin mediates the regeneration-promoting effects. Studies using knockout neurons showed that cofilin and ADF, which can have redundant functions, both contribute to the reversal of the developmental axon extension program established in adult sensory neurons by a conditioning lesion. Overexpression of cofilin also promotes the extension of axons on chondroitin sulfate proteoglycans (CSPG) and Nogo-A, extracellular inhibitors of axon growth. Thus, cofilin is emerging as an important regulator of both the developmental switch in sensory axon growth pattern and the regenerative competency of axons.

Regeneration of axons is impaired by the presence of multiple extracellular inhibitory signals. While a full discussion of the mechanisms mediating the inhibition of axon regeneration by these signals is beyond the scope of this review, the reader is directed toward reviews on these issues (Schwab and Strittmatter, 2014; McKerracher and Rosen, 2015; Tran et al., 2018). However, in congruence with the main themes addressed herein, these inhibitors of axon regeneration decrease growth cone complexity ranging from full collapse (loss of all filopodia and lamellipodia) to causing the growth cone to act in a “dystrophic’ manner, characterized by attenuated formation and elaboration of lamellipodia and filopodia (Li et al., 1996; Tom et al., 2004; Kurihara et al., 2012; Manns et al., 2014; Sainath et al., 2017). As noted in the first paragraph of this section, axons can extend in the absence of growth cones, albeit in a context-dependent manner. Thus, the mere attenuation of actin filament dynamics by inhibitory signals is not likely to fully explain their inhibitory effects. A major aspect of actin filament biology in growth cones is to interact with the mechanoenzyme myosin II to generate both pulling forces through substratum attachment, promoting the advancement of the growth cone at a normal rate, and to attenuate the forward advance of microtubules required for axon extension (Bridgman et al., 2001; Burnette et al., 2008; Schaefer et al., 2008). Inhibition of myosin II activity decreases the rate of sensory axon extension on laminin-coated substrata, which require myosin II-dependent substratum attachment, but promotes axon extension on a polylysine-coated substratum, which does not require myosin II-dependent attachment, by promoting the advancement of microtubules in growth cones (Turney and Bridgman, 2005; Ketschek et al., 2007). Inhibition of myosin II also promotes the ability of axons to extend on CSPG, inhibitors of axon extension and regeneration, and to cross from a permissive substratum onto a CSPG-coated substratum (Hur et al., 2011; Yu et al., 2012). *In vitro*, semaphorin 3A induces sensory growth cone collapse, followed by axon retraction (Gallo, 2006; Brown et al., 2009). The induction of growth cone collapse is independent of myosin II, but the ensuing retraction requires myosin II activity. Although growth cones collapse after treatment with semaphorin 3A, the axon shaft responds by developing a novel cytoskeletal organization consisting of actin filament bundles, which likely serve as the substratum for the myosin II-based force generation that drives subsequent retraction (Gallo, 2006; Brown and Bridgman, 2009). These studies indicate that to understand how axon extension-inhibiting signals impact the function of the actin filament cytoskeleton, it is necessary to consider not just the levels of actin filaments, but also the organization of actin filaments and their distribution since form and function are linked. Consistent with this notion, RhoA is a GTPase that regulates the dynamics and organization of the actin cytoskeleton and concurrently promotes myosin II activity (Ridley, 1997; Somlyo and Somlyo, 2000; Dupraz et al., 2019). RhoA is activated by and mediates, at least in part, the effects of a variety of axon extension/regeneration inhibitory signals (Fujita and Yamashita, 2014). Inhibition of RhoA signaling has axon growth-promoting effects *in vivo*, and *in vitro*, promotes axon extension on axon growth-inhibitory substrata. In the context of semaphorin 3A, RhoA drives the formation of the axonal actin filament bundles that are required for the subsequent retraction of the axon (Gallo, 2006; Brown and Bridgman, 2009). Furthermore, activation of the RhoA pathway inhibits cofilin, which results in the suppression of both the actin filament-based structures that generate contractile forces for axon extension and those that mediate the formation of filopodia and lamellipodia (Bamburg et al., 1999). The coordinated reorganization of the actin cytoskeleton, and the activation of myosin II, set the tip of the axon in a contractile state, which is functionally opposite to that required to promote extension and guidance. It will be of interest to further understand how regeneration-inhibiting signals impact the organization of actin filaments in the growth cone and axon, and how these organizational changes translate into inhibition of axon growth, beyond the mere decrease in actin filament content of the growth cone.

## Microtubules

The polymerization and transport of axonal microtubules are necessary mechanistic aspects of axon extension (Dent and Gertler, 2003). Within growth cones, the plus tips of microtubules undergo dynamic instability and the tips advance into the peripheral domain through polymerization. Short microtubule “seeds” undergo long-distance transport throughout the axon, and likely serve as the initial building blocks for the formation of longer microtubules through subsequent plus tip polymerization. Following polymerization of tubulin dimers into the microtubule lattice, alpha-tubulin undergoes multiple time-dependent post-translational modifications that reflect the length of time that the dimer has been incorporated into the microtubule lattice (e.g., acetylation and detyrosination), and can have functional consequences on microtubules and proteins that associate with microtubules (e.g., motor proteins; for a comprehensive review see Song and Brady, 2015).

Given the fundamental role of microtubules in axon elongation, they have been considered potential targets for promoting axon regeneration. Although polymerization of microtubules is the primary way that they promote axon extension, initial studies sought to determine the effects of pharmacological stabilization of microtubules on axon regeneration. Taxol is a drug that has concentration-dependent effects on microtubules (Singh et al., 2008). At low concentrations it attenuates plus tips dynamic instability, while at higher concentrations it can promote microtubule plus tip polymerization. At high concentrations taxol also stabilizes microtubules against a variety of depolymerization-inducing insults. Treatment of spinal cord-injured rats with taxol resulted in the promotion of axon regeneration (Hellal et al., 2011). Taxol treatment also promoted the regeneration of injured optic nerves (Sengottuvel et al., 2011). However, the enhanced axon regeneration cannot be ascribed exclusively to the direct effects of taxol on axonal microtubules. The treatment also alters aspects of scar formation and inflammation at the injury site (Hellal et al., 2011; Sengottuvel et al., 2011). Regardless of the multiple cellular sites of action, these studies determined that drugs that impact microtubule stability and dynamics may have therapeutic value in promoting axon regeneration. However, these drugs can lead to peripheral neuropathy, a potential major therapeutic caveat (Landowski et al., 2016; Tamburin et al., 2019). Furthermore, in contrast to the beneficial effects of taxol on CNS regeneration, treatment with taxol adversely affected regeneration in peripheral nerves (Hsu et al., 2017). Finally, microtubule dynamics are required for axon guidance. Therefore, taxol treatments may well impair the guidance of axons to appropriate targets, even if regeneration is promoted (Liu and Dwyer, 2014).

Microtubules in axons are subject to the action of microtubule-severing proteins (spastin, katanin, fidgetin; Matamoros and Baas, 2016). These proteins bind to and depolymerize microtubules, resulting in the formation of microtubule fragments. Gene dosage analysis in *Drosophila* indicates that normal axon regeneration requires normal levels of spastin expression, and spastin may promote regeneration through the regulation of endoplasmic reticulum (ER) repositioning to the tip of regenerating axons (Stone et al., 2012; Rao et al., 2016). The positioning of the ER at the axon tip was reduced in sensory axons growing on CSPG *in vitro*, emphasizing that ER is likely of significance to axon regeneration (Sainath et al., 2017). Whether CSPG affects ER positioning through spastin remains to be determined. Fidgetin is another microtubule-severing protein that upon downregulation, can promote axon extension *in vitro* on both permissive and inhibitory substrata (Austin et al., 2017). Depletion of fidgetin in adult sensory neurons *in vivo* promoted the entry of sensory axons into the spinal cord after a dorsal root crush injury (Austin et al., 2017). Collectively, these studies suggest that microtubule-severing proteins play a role in axon regeneration, likely through the regulation of the microtubule cytoskeleton and organelle positioning within axons, or through currently unclear additional functions of these proteins.

Kinesin 5 is a microtubule motor protein that attenuates the rate of axon extension (Myers and Baas, 2007). Pharmacological inhibition of kinesin 5 also promotes axon extension on CSPG *in vitro* and allows adult sensory axons to cross from a permissive substratum onto one containing CSPG (Lin et al., 2011). After a complete transection injury to the adult spinal cord *in vivo*, administration of the kinesin-5 inhibitor Monastrol, along with digestion of CSPG, promoted axon regeneration into a graft but did not result in functional improvement (Xu et al., 2015). Similarly, kinesin 12 has been reported to reduce both developmental and regenerative axon extension rates in zebrafish (Dong et al., 2019). In contrast, kinesin 1 mutant zebrafish exhibit impaired regeneration of peripheral axons (Ducommun Priest et al., 2019). Kinesin 1 mediates the anterograde transport of a variety of cargos, including mitochondria. The targeting of mitochondria to the tips of regenerating axons has emerged recently as a fundamental aspect of axon extension and regeneration (Smith and Gallo, 2018; Chamberlain and Sheng, 2019). The effects of manipulating kinesin 1 activity on regeneration may thus be attributable to dysregulation of axonal transport of required organelles. Dynein is the motor protein that mediates retrograde transport along axons (Olenick and Holzbaur, 2019). Zebrafish loss-of-function dynein mutants exhibit impaired peripheral axon regeneration that may be attributed to an impairment of microtubule stabilization during regeneration (Ducommun Priest et al., 2019). In addition to a role for dynein in regulating microtubule stability during regeneration, dynein-mediated retrograde transport also is involved in the regenerative response of axons following injury through retrograde nuclear signaling mechanisms (Hanz et al., 2003; Perlson et al., 2005; Ben-Yaakov et al., 2012). The above studies highlight that molecular motor proteins are emerging as potential targets for the promotion of axon regeneration.

Cytoplasmic alpha-tubulin that is available for polymerization into the microtubule lattice has a C-terminus tyrosine residue that is subsequently enzymatically removed after the tubulin is incorporation into the microtubule lattice (Fukushima et al., 2009). This results in the dynamic plus ends of microtubules exhibiting an enrichment in tyrosinated tubulin, while the tubulin that has been incorporated previously into the lattice of the microtubule has undergone detyrosination. Axon injury increases the levels of tyrosinated tubulin at the injury site (Hall et al., 1991; Mullins et al., 1994; Cho and Cavalli, 2012). Tubulin tyrosine ligase (TTL) is the enzyme that adds the C-terminal tyrosine to tubulin. In cultured adult sensory neurons, TTL is required for the injury-induced increase in tyrosinated tubulin levels, which in turn supports retrograde signaling that promotes axon regeneration (Song et al., 2015). Pharmacological inhibition of detyrosination *in vivo* also results in increased regeneration after sciatic nerve crush injury (Gobrecht et al., 2016).

Alpha-tubulin also undergoes time-dependent acetylation after it is polymerized into the microtubule lattice. The result is that the dynamic plus ends of microtubules have low levels of acetylated tubulin, while the main lattice of previously polymerized microtubules exhibits high levels of tubulin acetylation (Fukushima et al., 2009). The acetylation of tubulin correlates with but does not appear to causally contribute to, the stability (e.g., longevity) of microtubules (Perdiz et al., 2011; Song and Brady, 2015; Baas et al., 2016). Nevertheless, tubulin acetylation regulates a variety of microtubule-dependent processes in cells, including the promotion of axonal transport (Perdiz et al., 2011). Axon injury decreases tubulin acetylation levels in regeneration-competent peripheral axons but not central axons (Cho and Cavalli, 2012). Histone deacetylase 5 (HDAC5) acts as a tubulin deacetylase and mediates the injury-induced deacetylation. One report indicated that inhibition of HDACs impaired regeneration of peripheral sensory axons following nerve crush *in vitro* and *in vivo* (Cho and Cavalli, 2012). However, this finding was challenged by a subsequent study in which promoting acetylation by inhibiting HDAC5 or overexpressing the alpha-tubulin acetyltransferase (αTAT1) failed to promote sensory axon regeneration following sciatic nerve crush *in vivo* (Lin et al., 2017). Interestingly, in that study, axon extension was promoted by αTAT1 *in vitro*, but this was independent of its transferase activity. Thus, the issue of whether tubulin acetylation is involved in regulating axon regeneration would benefit from the continued investigation.

## Neurofilaments

The third and most abundant components of the neuronal cytoskeleton are the neurofilaments (NFs), which provide structural support and determine axon caliber (Hoffman et al., 1987). While abundant in axons, NFs are sparse in dendrites and neuronal cell bodies (Burton and Wentz, 1992). Within axons, these 10 nm intermediate filaments are arranged in parallel arrays spaced by side chains that extend perpendicular to the filament core. In immature CNS neurons, NFs self-assemble into heteropolymers of the light and medium molecular mass NF proteins (NF-L and NF-M, respectively), and α-internexin (Kaplan et al., 1990; Yuan et al., 2006). With maturation, the heavy molecular mass NF protein (NF-H) gradually becomes incorporated into the NFs (Carden et al., 1987). NF subunits share a similar structure including a variable N-terminal head domain, a C-terminal tail of varying length, and a conserved central α-helical rod region, which mediates self-assembly *via* coil-coil interactions (Yuan et al., 2017). Both the head domain and C-terminal are subject to post-translational modifications, including glycosylation and phosphorylation. Extensive phosphorylation of lysine-serine-proline repeats within the C-terminal tail is particularly important in conveying stability to the filament. NF-H contains over 40 of these repeat motifs, and this is associated with enhanced NF stability and loss of dynamism as the neuron matures. Remarkably, heavily phosphorylated NFs have an *in vivo* half-life estimated at approximately 55 days (Nixon and Logvinenko, 1986). Although, NFs are often considered obligate heteropolymers, NF-L, α-Internexin, and a 5th NF subunit found exclusively in the PNS, type III peripherin, are capable of forming homopolymeric filaments under some conditions (Carter et al., 1998; Beaulieu et al., 1999; Yuan et al., 2006).

The role of NFs in axon regeneration remains unclear. Immediately after injury, NF expression is suppressed but recovers among neurons undergoing successful regeneration (Hoffman and Cleveland, 1988; Muma et al., 1990; McKerracher et al., 1993; Jacobs et al., 1997; Gervasi et al., 2003). Indeed, during active regeneration, NF subunit mRNA levels often exceed levels observed in uninjured neurons and maybe more translationally active (Tesser et al., 1986; Gervasi et al., 2003; Ananthakrishnan and Szaro, 2009). The purpose of this transient reduction in NF expression is unknown but may serve to enhance cytoskeleton dynamics in the injured axon, particularly the infiltration of tubulin into the growth cone (Oblinger et al., 1989; Tetzlaff et al., 1996). Alternatively, it has been suggested that changes in NF expression after injury may represent an attempt at the recapitulation, albeit unsuccessful among CNS neurons, of the embryonic axon growth program, where triplet NF proteins are suppressed in favor of other intermediate filaments (Szaro and Strong, 2010). Interestingly, NF-L knockout mice, which co-experience precipitous declines in NF-M and NF-H levels, develop normally but experience impaired PNS axon regeneration after sciatic or facial nerve crush (Zhu et al., 1997). Similarly, inhibiting NF expression in other models reduces regeneration efficiency but does not fully abolish outgrowth. Notably, in dissociated embryonic frog (*Xenopus laevis*) spinal cord culture, inhibition of NF-M reduced the time neurites spent actively growing but did not alter outgrowth velocity (Walker et al., 2001). In lampreys, which undergo robust axon regeneration after a complete SCI, *in vivo* inhibition of the lamprey NF-M-like homolog, NF180, reduced the number of axons regenerating 5 mm beyond the SCI at 4 and 9 weeks post-injury (Zhang et al., 2015). However, regeneration was not grossly inhibited and the initial axon retraction after injury was unaltered. Notably, NF-H overexpression attenuated neurite outgrowth in differentiated Nb2a/d1 neuroblastoma cells (Boumil et al., 2015). This was likely the result of increased NF stability since outgrowth was unaffected in mutants lacking the NF-H C-terminal. Interestingly, inducing expression of vimentin, another intermediate filament expressed predominately by neural precursors, promoted neurite outgrowth in these same cells (Dubey et al., 2004). Ultimately, the role of the NF subunits in promoting or inhibiting regeneration may be due, in part, to their net effects on cytoskeletal dynamics, with excess stability unfavorable to outgrowth.

Although *in vitro*, growth cones may form within hundreds of micrometers from the neuronal cell body, *in vivo* growth cones often form centimeters, or even meters away. Thus, the question arises, how do the NFs arrive in the distal axon? NFs are actively transported, bidirectionally, along with the microtubule network (Helfand et al., 2003; Uchida and Brown, 2004), and it was originally believed that NFs were synthesized in the soma, then anterogradely transported, whether as individual subunits or partially assembled, to where they were needed, with excess NF subunits being bulk degraded in the axon terminal. However, increasingly convincing evidence suggests that NF subunits also are synthesized locally within the axon. Early studies noted that the NF assembly in the distal axon appeared independent of NF subunit synthesis in the soma (Tetzlaff and Bisby, 1989). More recently, studies have profiled NF mRNAs in the axon and demonstrated that they are preferentially enriched in the growing tips (including growth cones) of axons (Zheng et al., 2001; Lee and Hollenbeck, 2003; Baraban et al., 2013; Wang et al., 2014; Jin et al., 2016). Local NF protein synthesis has been demonstrated *in vitro* and EM analysis suggests synthetic capability is also present in axons *in vivo* (Zheng et al., 2001; Lee and Hollenbeck, 2003; Jin et al., 2016). Interestingly, *in vivo* evidence from lampreys suggests that NF mRNAs are enriched selectively in actively elongating axon tips, suggesting that local NF synthesis may contribute directly to regeneration (Jin et al., 2016).

## Growth Cones vs. Neurofilament-Packed Axon Tips

**Figure 3:**
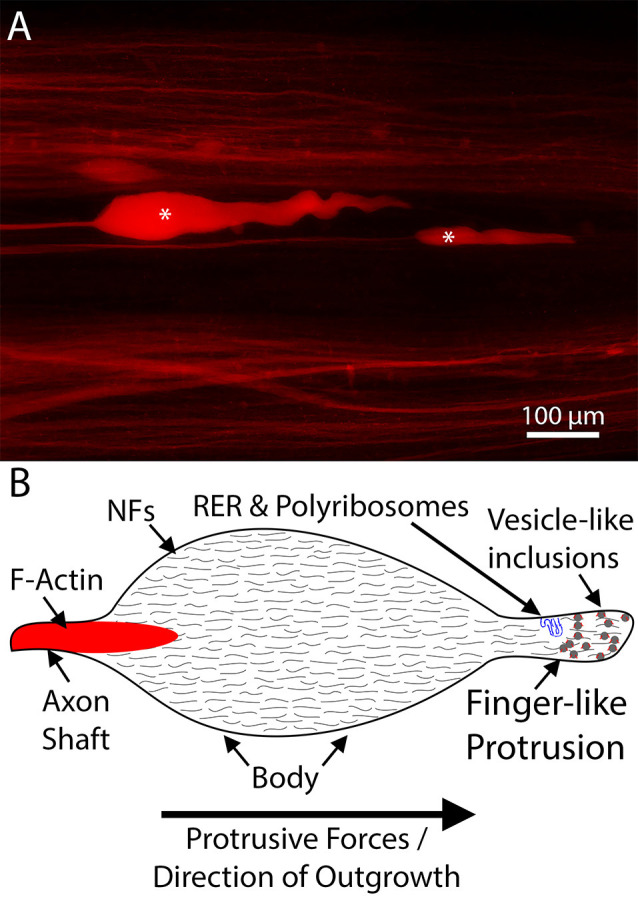
An example of lamprey neurofilament-packed axon tips. **(A)** Representative dye-labeled lamprey axon tips (*) in cleared wholemount spinal cord, 10 days post-spinal cord injury (SCI). Note the absence of filopodia and lamellipodia. **(B)** Schematic of a typical regenerating lamprey axon tip. These tips contain little F-actin but are packed with neurofilaments (Lurie et al., 1994; Jin et al., 2009). Emerging from the distal axon shaft, the tip consists of an enlarged body and a finger-like protrusion, which in some tips contains structures resembling rough endoplasmic reticulum (RER; Jin et al., 2016). In actively growing tips, the distal region of the tip is often filled with numerous vesicle like-inclusions decorated by F-actin (Jin et al., 2009). Note, the shapes of the tips vary *in vivo*, likely relating to whether the tip is elongating or retracting, but consistently lack filopodia and lamellipodia.

Due to their association with cytoskeletal stability, NFs were initially not believed to contribute significantly to growth cone dynamics. Indeed, early studies suggested that bulk accumulation of NFs in axon terminals was prevented by calcium-mediated proteolysis (Roots, 1983). Nevertheless, it has been shown that a dynamic population of NFs resides within the central region of growth cones (Tennyson, 1970; Chan et al., 2003). Moreover, *in vitro* experiments have demonstrated that compared to the trailing axon shaft, growth cones were more highly enriched in newly synthesized NF subunits (Chan et al., 2003). These newly synthesized subunits were believed to participate in regional NF formation, to provide structural support to the elongating axon. However, evidence from lower vertebrates, especially lampreys, raises the possibility that NFs play a more direct role in axon outgrowth. Lamprey CNS axons, despite their impressive ability to regenerate, do not form canonical growth cones after injury (Lurie et al., 1994; Figure 3). Their relatively simple axon tips lack filopodia and lamellipodia and contain little F-actin (Hall et al., 1997; Jacobs et al., 1997). Instead, they are densely packed with NFs, whose expression patterns are correlated with regeneration (Lurie et al., 1994; Jacobs et al., 1997). Thus, an alternate mechanism for axon elongation has been hypothesized in this model. Rather than canonical actin-microtubule treadmilling, protrusive forces from NF assembly have been postulated to drive axon outgrowth (Zhang et al., 2005). Although intriguing, evidence supporting this hypothesis as a general mechanism of axon elongation has been slow to accumulate. In part, this may be due to the difficulty of imaging regenerating axons *in vivo*, forcing many studies of mammalian growth cones to use *in vitro* models. The resulting short-distance neurite outgrowth observed in these systems may be mechanistically distinct from sustained long-distance regeneration *in vivo*. Support for this hypothesis can be found in embryonic DRG cultures, in which inhibiting F-actin polymerization with cytochalasin B, collapsed the filopodia and lamellipodia but did not abolish neurite outgrowth—although subsequent reports have questioned whether F-actin assembly was truly abolished or merely severely reduced (Marsh and Letourneau, 1984; Letourneau et al., 1987; Chia et al., 2016). Of interest, even in the regenerating lamprey axons that lack filopodia and lamellipodia, and have little F-actin, the growing tips contain numerous vesicle-like inclusions, and these appear to be surrounded by a layer of F-actin (Jin et al., 2009). These inclusions may provide materials to extend the axolemma during regeneration, in a membrane recycling process that involves sub-axolemmal F-actin (Prager-Khoutorsky and Spira, 2009; Bloom and Morgan, 2011).

The question arises whether the absence of typical growth cones in severed lamprey spinal cord axons represents a general difference between embryonic axons and axons in cell cultures on the one hand, and regenerating CNS axons *in vivo* on the other, or merely a peculiarity of lamprey neurons. Unfortunately, this is difficult to test, because lamprey neurons have proven challenging to culture reliably for more than a few hours. Nevertheless, in those *in vitro* studies that had some success, neurite tips assumed varying morphologies (Ryan et al., 2007; Pale et al., 2013). While some neurites had bulb-like endings resembling lamprey axon tips *in vivo*, others developed typical-looking growth cones, with structures that look like filopodia and lamellipodia. The molecular/cytoskeletal contents of these growth cones have not been studied, and it also is unlikely that the cultured neurons include the large reticulospinal neurons typically imaged *in vivo*. Nevertheless, those results support the hypothesis that the morphological differences between growth cones and growing CNS axon tips in the intact animal reflect in part the differences between *in vitro* and *in vivo* environmental conditions, associated with a developmental loss of the ability to form growth cones in most post-natal neurons. If standardized, an *in vitro* lamprey neuron model would be very useful to unravel the conditions determining the formation of canonical growth cones or neurofilament-packed tips and, critically, more fully elucidate the molecular mechanisms underlying neurofilament-associated axon outgrowth. The distinction could be important if the growth cone represents the anatomical substrate for axon guidance over short distances, perhaps more akin to collateral sprouting in the CNS, often referred to as “axonal plasticity,” whereas the kind of long-distance regeneration that would be needed to restore connections in many instances of CNS injury might require a different, more sustained mechanism of axon elongation.

## Discussion

To recover lost function, injured axons must regenerate. However, in the mammalian CNS, unlike the PNS or the CNS of lower vertebrates, regrowth stalls. In part, this failure is due to inefficient cytoskeletal dynamics at the axon tip. Elucidating the mechanisms by which cytoskeletal rearrangements mediate axon outgrowth is essential to identifying therapeutic targets to promote sustained regeneration after injury. Despite substantial advances in our understanding of these mechanisms, many questions have yet to be fully answered. These questions include the roles of protein transport and local synthesis in providing cytoskeletal components to distal axon regions, how the balance between filament stability and dynamism shapes outgrowth, whether alternate mechanisms to actin treadmilling may mediate axon extension during regeneration, and how the mechanisms underlying regeneration of axons in the injured CNS differ from those that mediate collateral sprouting by neighboring spared axons.

It is clear that the growth cone and its actin filament cytoskeleton are critically important to axon growth in early development, and are implicated in axon growth in the CNS after injury. However, as noted above, the growth cone is not essential to axon elongation in all circumstances, and it is not clear that growth cones could underlie longer-distance regeneration of axons after injury in the mature CNS, as seen after spinal cord transection in the lamprey (Lurie et al., 1994; Jacobs et al., 1997; Jin et al., 2009). Thus, environmental factors that inhibit axon growth in mammals (e.g., Nogo), and trigger growth cone collapse *in vitro*, maybe suppressing collateral sprouting rather than true axon regeneration *in vivo* (Lee et al., 2009). This is particularly relevant to mammalian studies since it often is difficult to distinguish regeneration of injured axons from collateral sprouting by uninjured neighboring axons. Thus, cautious investigators often use the more general term “axonal plasticity” (Blesch and Tuszynski, 2009). While canonical growth cones and neurofilament-packed, vesicle-containing growing tips may represent entirely distinct mechanisms of axon regeneration, it also may be possible that they represent opposite ends of a spectrum of regenerating axon morphologies, depending on neuron-intrinsic and environmental cues, and the stage of outgrowth (e.g., pathfinding vs. elongation).

## Author Contributions

WR, GG, and MS contributed to the literature review and writing of the manuscript. All authors read and approved the submitted version.

## Conflict of Interest

The authors declare that the research was conducted in the absence of any commercial or financial relationships that could be construed as a potential conflict of interest.
